# How leaders restrict employees’ deviance: An integrative framework of interactional justice and ethical leadership

**DOI:** 10.3389/fpsyg.2022.942472

**Published:** 2022-08-09

**Authors:** Jinsong Li, Haoding Wang, Yahua Cai, Zhijun Chen

**Affiliations:** College of Business, Shanghai University of Finance and Economics, Shanghai, China

**Keywords:** ethical leadership, moral disengagement, deviance, social cognitive theory, leader interactional justice

## Abstract

Past research illustrated that leaders could restrict followers’ deviance by reinforcing social norms of appropriate behaviors. Nevertheless, we submit that this understanding is incomplete without considering the effects of leaders on followers’ self-sanctions given that most undesirable behaviors are controlled internally. This research argues that interactional justice is an effective strategy for leaders to enhance followers’ self-sanctions. Leaders’ interactional justice provides personalized information and dyadic treatment that indirectly reduce employees’ deviance by restraining followers’ moral disengagement. Besides, this study examines the social sanction role of ethical leadership. Ethical leaders highlight the importance of adherence to collective norms, which influence the relationship between followers’ moral disengagement and deviance. By identifying the different pathways via which they influence followers’ moral disengagement, we integrate interactional justice and ethical leadership into one theoretical framework. Our predictions are supported by data analyses of 220 samples from a multi-wave and -source field study. This integrative framework contributes to a comprehensive understanding of how leaders restrict employees’ deviance.

## Introduction

Given its harmful purposes and norm-violation nature, employee deviance is especially problematic for organizations, which costs organizations billions of dollars per year in lost productivity and other expenses (Berry et al., 2007). Hence, one significant role of managers is to restrict employees’ deviance, keeping followers’ activities in line with organizational norms and objectives (Davis and Rothstein, 2006). According to prior research, managers can curb employees’ deviance by directly highlighting the importance of appropriate behaviors and punishing the employees who violate the organizational norms (Lord et al., 2017; Kleshinski et al., 2021). The most well-known example is ethical leadership. Ethical leaders demonstrate normatively appropriate conduct and promote such behaviors through communication and reinforcement, which had been approved as an effective way to restrain employees’ deviance and other misbehaviors (Brown and Treviño, 2006; Bedi et al., 2016; Peng and Kim, 2020).

However, although existing research showed that leaders could restrict employees’ deviance through external social sanctions like punishment or even dismissal (Bandura, 2002; Mayer et al., 2010), little is known about how leaders influence employees’ self-sanctions. This is a significant omission since most misconducts are internally refrained by individuals’ self-sanctions, such as negative emotions and self-reproof (Bandura, 1991, 1999). Thus, exploring the behavioral strategies leaders can adopt to enhance followers’ self-sanctions is crucial, which enriches a comprehensive understanding of how leaders restrict followers’ deviance.

In this research, we submit that leaders’ interactional justice can enhance followers’ self-sanctions by restraining followers’ moral disengagement. Moral disengagement refers to cognitive strategies for deactivating self-sanctions, such as justification and displacing responsibility, which may lead to various undesirable behaviors (Bandura, 2011). This research proposes that followers’ moral disengagement will be limited by leaders’ interactional justice because leaders’ interactional justice provides respect and sincerity that preserve followers’ sense of dignity, which is essential for followers to hold moral self-sanctions (Bandura et al., 1996; Bies, 2001; Johnson et al., 2010). As a result, followers’ self-sanctions restrain deviance when leaders limit followers’ moral disengagement by interactional justice. We choose interactional justice rather than procedural and outcome justice because only interactional justice directly highlights respect in relationships, whereas procedural and outcome justice mainly focuses on the issues of interests (Brockner et al., 2007; Scott et al., 2009; O’Reilly et al., 2016).

**FIGURE 1:**
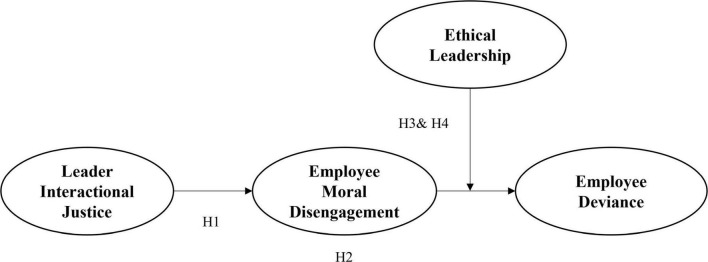
The hypothesis model.

Moreover, we argue that leaders can simultaneously promote social sanctions and followers’ self-sanctions to restrict employees’ deviance further. Leaders’ interactional justice constrains followers’ deviance by limiting their disengagement. Ethical leadership, which reflects social sanctions, can prevent followers from translating moral disengagement into deviance (Brown and Treviño, 2006). Ethical leadership underscores the adverse consequences of norm violations by promoting and reinforcing behavioral rules (Barnett and Vaicys, 2000; Bandura, 2011). Thus, even though employees escape personal sanctions via moral disengagement, they are reluctant to partake in misconduct when social sanctions are salient (Bazerman and Sezer, 2016; Hirsh et al., 2018). As a result, ethical leadership mitigates the positive effect of follower moral disengagement on deviance. By integrating the approaches of interactional justice and ethical leadership, leaders can reduce the employees’ moral disengagement and mitigate the adverse effects once moral disengagement arises. Our theoretical model is shown in Figure 1.

We contribute to the behavioral ethics and leadership literature in three ways. First, we argue that leaders’ interactional justice is a critical way of enhancing followers’ self-sanctions, and we integrate interactional justice approaches and ethical leadership approaches by revealing their different mechanisms of influencing followers’ moral disengagement. Second, by elucidating how ethical leadership influences the relationship between followers’ moral disengagement and deviance, this research enriches the understanding of the effects of leaders on employees’ moral disengagement (Newman et al., 2020). Finally, beyond learning-, exchange-, and emotion-based explanations that focus on its direct impact (Peng and Kim, 2020), this research examines the social sanction effect of ethical leadership, wherein ethical leadership plays a role as a moderator in the relationship between employees’ moral disengagement and their deviance.

## Theoretical background and hypotheses

### Social cognitive theory of moral thought and action

According to social cognitive theory, misconducts are regulated by two sanctions: social sanctions and internalized self-sanctions (Bandura, 1991, 1999). In this regard, social sanctions restrain transgressive conduct via adverse social consequences (e.g., punishment), and self-sanctions influence individuals’ sense of self-images (e.g., self-condemnation). Accordingly, there are two behavioral approaches by which leaders restrain followers’ deviant behaviors. The first is promoting social regulation, wherein leaders directly promote collective norms of appropriate behaviors through role modeling, communication, and reinforcement (Brown and Treviño, 2006). The second is promoting employees’ self-regulation, in which leaders aim to enhance followers’ moral self-regulatory system, e.g., by developing employees’ self-regulatory competence (Bandura, 1991; Owens et al., 2019) and facilitating selective activation and disengagement of moral self-regulation (Moore et al., 2019).

This research integrates these two approaches by theorizing the distinct pathways for influencing employees’ moral disengagement. First, social realities affect the operation of the moral self-regulatory system, that is, leaders can take actions to reduce employees’ moral disengagement (Bandura, 1999). Given that moral disengagement is an inherently interpersonal phenomenon (Johnson and Buckley, 2015), the activation or deactivation of employees’ moral disengagement is most likely to occur in leader-member dyadic interactions. Thus, this study submits that leaders can curb employees’ moral disengagement via interactional justice behaviors. Being treated truthfully and respectfully by leaders reduces the opportunity and motivation for employees to apply strategies of moral disengagement (Johnson et al., 2010; Bies, 2015).

Second, moral conduct is simultaneously regulated by personal and social sanctions because (un)ethical behaviors produce both self-evaluative reactions and social effects (Bandura, 1991). Accordingly, we argue that the behavioral approaches arising from ethical leadership could prevent follower moral disengagement from turning into deviance. Ethical leadership approaches mainly focus on promoting social regulation, which underscores the importance of normatively appropriate behaviors (Mayer et al., 2012; Ng and Feldman, 2015). Severe social consequences of norm violation would prevent employees’ misconduct, even though they gain self-approval via moral disengagement. In summary, by theorizing the difference in how interactional justice and ethical leadership influence employees’ moral disengagement, this research integrates two behavioral approaches into a framework based on social cognitive theory.

### Leaders’ interactional justice and employee moral disengagement

Interactional justice refers to the fair treatment people receive in interpersonal relationships (Bies and Moag, 1986). Here, fair treatment means that decision-makers treat people with respect and honesty (i.e., interpersonal justice) and explain the rationale for decisions in a timely, open manner (i.e., informational justice, Bies, 2001). Leaders’ interactional justice is valued by most employees (Liang et al., 2021), and as such it generates various positive attitudes and behaviors including higher trust and leader-member exchange (LMX) (Lam et al., 2013), improved employee identification and commitment (Colquitt et al., 2013), and heightened work performance and extra-role behaviors (e.g., OCB, He et al., 2017). Moreover, numerous scholars highlight the moral nature of interactional justice because such behaviors are based on moral-laden principles (truth and human dignity, Bies, 2015; O’Reilly et al., 2016; Barclay et al., 2017). Nevertheless, how leaders’ interactional justice fosters followers’ ethical action is largely unexamined.

Drawing on social cognitive theory, we propose that leaders’ interactional justice could restrain followers’ deviance by curbing cognitive strategies related to moral disengagement (Bandura, 1991). On the one hand, leaders acting according to the principle of truth treat their subordinates with sincerity, explaining decisions to followers in a timely, open, and truthful manner (Bies, 2001). Such explanations contribute to a clear responsibility boundary. Thus, it is difficult for employees to displace or diffuse responsibility even if the decision is undesirable (Holt et al., 2021). For example, when faced with unfavorable outcomes, employees treated in accordance with leaders’ interactional justice are more inclined to experience inward-focused negative emotions rather than blame the leaders (Barclay et al., 2005).

On the other hand, the principle of human dignity refers to leaders acting politely and respectfully (Bies, 2001). When leaders behave with courtesy and treat followers as respectable persons, employees tend to value human rights and dignity as well (Leung et al., 2004; Ambrose et al., 2013) such that leaders’ interactional justice reduces the tendency for employees to disregard others’ feelings (Moore et al., 2019). Accordingly, followers treated with interactional justice by leaders are less likely to rationalize harm to others or dehumanize other people (Bandura, 1991). This argument is akin to the consensus of existing fairness research that followers would learn from leaders’ interactional justice and be kind and polite to co-workers and other people (Skarlicki et al., 2016). In summary, we expect leaders’ interactional justice to reduce followers’ moral disengagement.

**Hypothesis 1:** Leaders’ interactional justice is negatively related to employees’ moral disengagement.

### Mediating role of moral disengagement

Existing research shows that moral disengagement is related to a variety of undesirable outcomes, such as unethical decision-making (Detert et al., 2008), deviant behavior (Johnson and Buckley, 2015), and social undermining (Lee et al., 2016), among others (Newman et al., 2020). The theoretical underpinning of these studies is that moral disengagement deactivates individual internal self-sanctions (Bandura, 1991, 1999). Thus, employees with high moral disengagement gain self-approval to violate norms of the organization or society, evoking selfish mindsets that care less about rules and others (Johnson and Buckley, 2015). Therefore, when leaders’ interactional justice reduces followers’ moral disengagement, followers’ self-regulatory systems can prevent them from engaging in deviance that harms others. This is because leaders’ interactional justice directly suppresses the cognitive mechanisms of moral disengagement. When leaders adhere to the rule of interpersonal justice, treating their followers as respectable people, followers are less likely to disregard others’ feelings and engage in deviant behavior that harms others. In addition, leaders’ informational justice can curb followers’ tendency to disregard their responsibility, which can prevent followers from partaking in deviance to fulfill selfish needs. To sum up, we suggest the existence of mediating effects of moral disengagement on the relationship between interactional justice and deviance. Leaders’ interactional justice mitigates subordinate deviance by decreasing their moral disengagement.

**Hypothesis 2:** Employees’ moral disengagement mediates the relationship between leaders’ interactional justice and employees’ deviance.

### Ethical leadership as a moderator

Ethical leadership refers to a role model that “demonstrates normatively appropriate conduct through personal actions and interpersonal relationships and promotes such conduct to followers through two-way communication, reinforcement, and decision making” (Brown et al., 2005, p. 120). Ethical leadership theory is rooted in social learning theory (Bandura, 1977; Brown et al., 2005). Ethical leaders enhance ethical climates such as rules and caring climate that promote behavioral norms. From these norms, employees accept and learn how to do things in the morally right way (Pagliaro et al., 2018). Moreover, as moral people, ethical leaders demonstrate moral virtues like honesty, accountability, and trustworthiness. These qualities are essential for a positive leader-member relationship, which promotes mutual trust and obligations that positively impact followers’ moral behaviors (Peng and Kim, 2020). Ethical leadership is also beneficial for helping followers develop high levels of organizational identification (Niu et al., 2022), leading to positive employee attitudes and behaviors (Barattucci et al., 2020).

Although there is a broad consensus that ethical leadership can shape employees’ normative behavior, past research mainly focused on its direct effect (Peng and Kim, 2020). This is because previous studies are predominately based on social learning theory that ethical leaders are role models that teach employees the appropriate behaviors (Brown et al., 2005). However, ethical leadership includes multiple components such as moral virtues, moral management, and appropriate conduct that indicate multiple mechanisms (Liu et al., 2020). As more researchers call for expanding the understanding of ethical leadership’s various effects (Koopman et al., 2019; Wu, 2021), this research proposes the moderating effects of ethical leadership, highlighting ethical leaders as moral managers who provide social sanctions of deviance. Namely, ethical leadership can prevent employees’ moral disengagement from turning into deviance by indicating severe social consequences of violating moral norms. This argument builds on the tenets of social cognitive theory that (un)ethical actions are regulated by the interplay between personal and social sanctions (Bandura, 1991, 1999). The relative strength of social censure and self-approval determines whether the behavior will be restrained or expressed (Bandura, 1991).

Ethical leadership promotes social sanctions of misconduct, given that ethical leaders explicitly underscore the importance of moral codes and promote it with multiple leadership practices such as rewards, discipline, and communication to hold employees accountable to those codes (Brown and Treviño, 2014). Specifically, ethical leaders will penalize subordinates for violating moral rules, formally or informally. Violating the norms valued by ethical leaders runs the risk of losing multiple opportunities, such as opportunities for advancement or other rewards for upholding moral frameworks (Brown and Treviño, 2006). Explicit reward and punishment schemes will catch the attention of employees. Thus, ethical leadership provides clear social signals that misconduct is intolerable in the organization, enhancing employees’ sense of the “cost” of engaging in deviance (Hirsh et al., 2018). As a result, even if employees could escape self-sanction through moral disengagement, a high level of ethical leadership will restrain their deviance by emphasizing social sanctions. Previous studies have provided evidence for this argument (Mayer et al., 2010; Kuenzi et al., 2020). For instance, a strong ethical climate would prevent customer-directed sabotage even if employees have devalued the customer (Huang et al., 2019), and morally disengaged salespersons can temper counterproductive behavior when interacting with ethical leaders (Seriki et al., 2020). In summary, we submit that ethical leadership will mitigate the positive relationship between employees’ moral disengagement and their deviance.

**Hypothesis 3:** Ethical leadership moderates the relationship between employees’ moral disengagement and deviance such that this relationship is positively significant when ethical leadership is low and absent when ethical leadership is high.

Together, we expect a moderated mediation relationship. On the one hand, under high ethical leadership, the indirect effect of leaders’ interactional justice on deviance is weaker (Brown et al., 2005). On the other hand, when ethical leadership is low, leaders’ interactional justice could influence deviance by preventing employees from disengaging self-sanctions, indicating an indirect effect on follower moral decision-making through reducing moral disengagement (Bandura, 1999; Bies, 2001, 2015).

**Hypothesis 4:** Ethical leadership moderates the indirect effect of leaders’ interactional justice and deviance via moral disengagement such that this relationship is weaker when ethical leadership is high rather than low.

## Materials and methods

### Sample and procedures

Data were collected from a manufacturing enterprise in southeast China. We gained the approval of the firm’s CEO and met with the director of the Human Resources (HR) department. With the support of the HR department, we conducted an on-site survey using paper questionnaires. All subjects were gathered in meeting rooms when they participated in the survey. Participators were mainly from functional units such as quality control, marketing, HR, and product design. Before the survey, members of the research team clearly stated the academic purpose of the investigation; further, we applied a unique identifier code for each questionnaire and provided anonymous envelopes. We stayed on-site to answer questions and directly received the completed questionnaires during the survey. After the survey, each participant received a small gift (approximately 3 USD).

We collected paired data in three waves with 1-month time lag. One-month time lag is a widely accepted period to alleviate the CMV problem and ensure a low sample loss rate (Siemsen et al., 2010), which was widely applied in justice and ethics research (Lee et al., 2016; Liu et al., 2020). In the first-wave survey (T1), we asked employees to report their perception of leaders’ interactional justice and basic demographic information. At Time 1, we received 293 questionnaires. One month later, in the second-wave survey (T2), we asked the same employees to report their perception of ethical leadership and moral disengagement. We distributed the questionnaires to 281 employees in Time 2 and received 253 (86% response rate) completed samples. One month later, in the last-wave survey (T3), we instructed the supervisors to report the deviance of the focal employees. Questionnaires were distributed to 46 supervisors, and we gathered 220 employee questionnaires and 39 leader questionnaires (75% response rate of employees and 84% response rate of leaders). Regarding demographic characteristics, 59.5% of the sample were male, and the average age was 32.39 years (*SD* = 7.70). The average work tenure was 6.56 years (*SD* = 4.66).

### Measures

We measured all variables in our study with established scales. We translated the survey from English to Chinese that followed the translation and back-translation procedure (Brislin, 1980).

#### Interactional justice

Employees completed the 9-item interactional justice scale developed by Erdogan et al. (2006). Sample items included “When decisions are made about my job, my manager treats me with kindness and consideration,” and “When decisions are made about my job, my manager provides sufficient justification” (1 = “strongly disagree” to 5 = “strongly agree”; α = 0.90).

#### Ethical leadership

We used the 10-item ethical leadership scale from Brown et al. (2005). Sample items included “My supervisor discusses business ethics or values with employees,” and “My leader sets an example of how to do things the right way in terms of ethics” (1 = “strongly disagree” to 5 = “strongly agree”; α = 0.94).

#### Moral disengagement

We measured moral disengagement in workplaces with an 8*-*item scale in accordance with Moore et al. (2012). Employees were asked about their own agreement to the items. Sample items included “People shouldn’t be held accountable for doing questionable things when they were just doing what an authority figure told them to do,” and “Some people have to be treated roughly because they lack feelings that can be hurt” (1 = “strongly disagree” to 5 = “strongly agree”; α = 0.90).

#### Organizational deviance

Supervisors were asked to evaluate deviance of their subordinates with a 14*-*item scale developed by Stewart et al. (2009). Sample items included those on individual deviance such as “Said something hurtful to someone at work” and organizational deviance such as “Falsified a receipt to get reimbursed for more money than they spent on business expenses” (1 = “never” to 5 = “always”; α = 0.92).

#### Control variables

Several control variables are included to rule out competing explanations. It is worth noting that we tested our model without control variables, and all results (patterns and levels of significance) are essentially the same. Prior research showed that neuroticism is a typical trait in those individuals who score high in this trait tend to use self-focused cognitive patterns and are likely to engage in misconduct (Barlett and Anderson, 2012). In this regard, we measured neuroticism with 12 items from the NEO Personality Inventory (Costa and McCrae, 1992). Sample items include “I often feel tense and jittery” and “I am really fearful of anxiety” (1 = “strongly disagree” to 5 = “strongly agree”; α = 0.81). We also included the demographic characteristics of the employees, namely, age, gender, work tenure, and work position.

### Analytical strategies

Because our data had a nested structure (employees were nested within different teams), we adopted a sandwich estimator to compute the standard error estimator and to correct for potential statistical dependence. Specifically, we applied multi-level path analysis to test our hypotheses with Mplus 8.3. To test the proposed mediating role of employees’ moral disengagement (as described in Hypotheses 1 and 2), we utilized parametric bootstrapping with 20,000 iterations to estimate the significance of indirect effects (Bauer et al., 2006). To facilitate interpretation of the findings as described in Hypothesis 4, we grand mean centered ethical leadership and followers’ moral disengagement to obtain unbiased estimates of the interaction effects (Hofmann and Gavin, 1998). Finally, we used moderated path analysis to calculate the conditional indirect effects of follower perceived leaders’ interactional justice on leader perceived followers’ deviance via followers’ moral disengagement at high (+1 SD) and low (−1 SD) levels of ethical leadership (Edwards and Lambert, 2007).

### Confirmatory factor analysis and discriminant validity

Table 1 presents the means, standard deviations, and correlations of variables in this study. Before testing our hypotheses, we examined the discriminant validity of our key constructs (i.e., leaders’ interactional justice, employees’ moral disengagement, ethical leadership, deviance) through confirmatory factor analysis (CFA). As shown in Table 2, the hypothesized four-factor model fits the data well [χ^2^(521) = 837.60, *p* < 0.01, CFI = 0.92, TFI = 0.92, RMSEA = 0.05, SRMR = 0.05], and is superior to any three-factor model. Furthermore, we examine the common method variance in our study. In terms of Harman’s single-factor test of major variables, we conducted the exploratory factor analysis for all items that showed three existing factors extracted with an eigenvalue greater than 1. The amount of explanatory variance was 64.20%, and the largest factor accounts for the explanatory variance of 34.55%. All results indicated that common method bias was not a pervasive problem in our study.

**TABLE 1:** Means, standard deviations, coefficient alphas, and correlations.

Variable	*Mean*	*SD*	1	2	3	4	5	6	7	8	9
(1) Age	32.39	7.70									
(2) Gender	0.40	0.49	0.24**								
(3) Tenure	6.56	4.66	0.69**	0.18**							
(4) Position	1.64	0.69	0.25**	–0.01	0.15**						
(5) Neuroticism	3.31	0.96	0.02	0.09	–0.10	0.03	(0.81)				
(6) Interactional justice	3.64	0.64	0.08	–0.02	0.07	–0.02	–0.10	(0.90)			
(7) Ethical leadership	3.58	0.78	–0.11	–0.19**	–0.10	0.03	–0.28**	0.33**	(0.94)		
(8) Moral disengagement	2.62	0.70	–0.06	0.06	–0.07	–0.07	0.10	–0.37**	–0.24**	(0.90)	
(9) Deviance	1.95	0.58	–0.09	0.05	–0.19**	0.06	0.23**	–0.25**	–0.31**	0.39**	(0.92)

*N* = 220. For Gender, 0 = male; 1 = female. For position, 1 = Frontline employees, 2 = Frontline managers, 3 = Middle managers.

*p < 0.05; **p < 0.01. Bracketed values on the diagonal are the Cronbach’s alpha value of each scale.

**TABLE 2:** Results of confirmatory factor analyses.

Model	χ^2^	*df*	Δχ^2^	TLI	CFI	RMSEA	SRMR
Four-factor model^a^	837.60	521		0.92	0.92	0.05	0.05
Three-factor model-1^b^	1551.94	524	714.37**	0.74	0.76	0.10	0.12
Three-factor model-2^c^	1601.65	524	764.05**	0.72	0.74	0.10	0.13
Three-factor model-3^d^	1416.37	524	578.77**	0.77	0.79	0.09	0.09
Three-factor model-4^e^	1586.67	524	749.07**	0.72	0.75	0.10	0.11

*N* = 220.

^*a*^Measurement model.

^*b*^Combining ethical leadership and interactional justice.

^*c*^Combining ethical leadership and moral disengagement.

^*d*^Combining interactional justice and moral disengagement.

^*e*^Combining interactional justice and deviance.

*p < 0.05, **p < 0.01.

Δ, change relative to the measurement model; CFI, Comparative Fit Index; TLI, Tucker–Lewis index; RMSEA, Root Mean Squared Error of Approximation; SRMR, standardized root-mean-square residual.

### Hypotheses testing

Table 3 presents the results of the path analysis. Hypothesis 1 proposes that leaders’ interactional justice has a negative effect on followers’ moral disengagement. Supporting Hypothesis 1, the results show that interactional justice exerted a significant effect on employees’ moral disengagement (γ = –0.36, *SE* = 0.08, *p* < 0.01), which exceeds the influence of ethical leadership (γ = –0.11, *SE* = 0.06, *p* = 0.06).

**TABLE 3:** Multilevel path analysis results.

	Moral disengagement	Deviance	
	Model 1	Model 2	Model 3
Intercept	4.50 (0.44)**	1.72 (0.48)**	1.64 (0.49)**
**Control variables**			
Age	0.00 (0.01)	0.00 (0.01)	0.00 (0.01)
Gender	0.03 (0.08)	0.02 (0.06)	0.04 (0.06)
Tenure	–0.01 (0.02)	–0.03 (0.01)	–0.02 (0.01)
Position	–0.07 (0.07)	0.08 (0.05)	0.09 (0.05)
Neuroticism	0.02 (0.04)	0.07 (0.04)	0.07 (0.05)
**Independent variable**			
Interactional justice	–0.36 (0.08)**	–0.05 (0.07)	–0.03 (0.07)
**Moderator**			
Ethical leadership	–0.11 (0.06)	–0.14 (0.05)**	–0.15 (0.05)**
**Interaction**			
Ethical leadership × Moral disengagement			–0.21 (0.06)**
**Mediator**			
Moral disengagement		0.25 (0.05)**	0.25 (0.05)**
Residual Variances	0.42**	0.24**	0.23**

*N* = 220. *p < 0.05; **p < 0.01. Standard errors are in parentheses.

Hypothesis 2 proposes that employees’ moral disengagement mediates the relationship between leaders’ interactional justice and employees’ deviance. The results show that, when regressing employees’ deviance on leaders’ interactional justice and employees’ moral disengagement, the relationship between moral disengagement and deviance is significant (γ = 0.25, *SE* = 0.05, *p* < 0.01), whereas the effect of leaders’ interactional justice on employees’ deviance is non-significant (γ = –0.05; *SE* = 0.07, *p* = 0.44). Further, the parametric bootstrapping with 20, 000 iterations results showed that the indirect effect of leaders’ interactional justice on employees’ deviance via employees’ moral disengagement was significant (γ = –0.09; *SE* = 0.03, *p* < 0.01; 95% CI = [–0.15, –0.04]). Therefore, Hypothesis 2 was supported.

**FIGURE 2:**
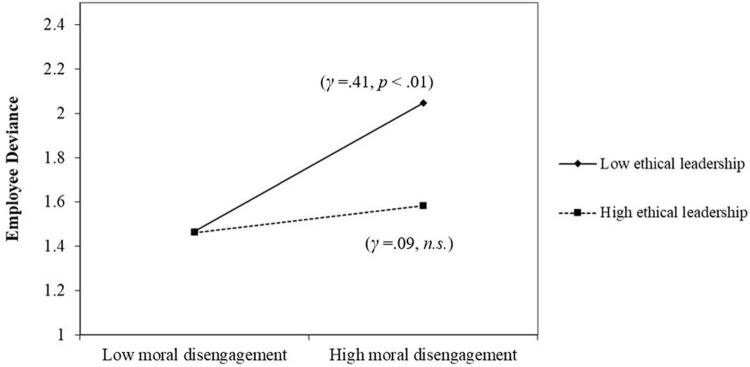
The moderating effects of ethical leadership on the relationship between moral disengagement and deviance.

In line with Hypothesis 3, the interactive effects of ethical leadership and followers’ moral disengagement on followers’ deviance were significant (γ = 0.21, *SE* = 0.06, *p* < 0.01). As shown in Figure 2, subsequent simple slope analysis showed that moral disengagement was positively related to deviance when ethical leadership was low (–1 *SD*, γ = 0.41, *SE* = 0.08, *p* < 0.01) but not when ethical leadership was high (+1 *SD*, γ = 0.09, *SE* = 0.06, *p* = 0.18). The difference between the two simple slopes was significant (γ = 0.33, *SE* = 0.08, *p* < 0.01). These results support Hypothesis 3.

Hypothesis 4 proposes a moderated mediation model. As shown in Table 4, parametric bootstrapping with 20,000 iterations showed that the indirect effect of leaders’ interactional justice on followers’ deviance via followers’ moral disengagement was significant when ethical leadership was low (γ = –0.15, *SE* = 0.05, *p* < 0.01, 95% CI [–0.25, –0.07]) but not when it was high (γ = –0.03, *SE* = 0.02, *p* = 0.21, 95% CI = [–0.08, 0.01]). The difference of these two indirect effects was significant (γ = 0.12, *SE* = 0.05, *p* < 0.01, 95% CI = [0.04, 0.22]). Taken together, these results support Hypothesis 4.

**TABLE 4:** Result of moderated mediating effect of ethical leadership.

	Interactional justice → Moral		
	disengagement → Deviance		
Moderator: Ethical leadership	γ	*s.e.*	95% CI
Conditional indirect effect			
High ethical leadership (+1 SD)	−0.03	0.02	[–0.08 to 0.01]
Low ethical leadership (–1 SD)	−0.15**	0.05	[–0.25 to –0.07]
Difference	0.12**	0.05	[0.04 to 0.22]

*N* = 220. Bootstrapping = 20,000.

## Discussion

Drawing on social cognitive theory, this research integrates the effects of leaders’ interactional justice and ethical leadership on employees’ moral cognition and behavior (Bandura, 1991). Our research supports the assumption that perceived leaders’ interactional justice significantly reduced followers’ deviance by mitigating followers’ moral disengagement. In addition, ethical leadership influenced the relationship between employees’ moral disengagement and deviance. Moral disengagement elicited deviance under low ethical leadership, and this relationship was not significant under high ethical leadership. These findings have important theoretical and practical implications.

### Theoretical implications

First, this research contributes to an enriched understanding of how leaders restrain employees’ deviance. A broad consensus is that leaders are responsible for curbing followers’ deviance (Kleshinski et al., 2021). However, most research focused on the social sanction role of leadership and omitted leaders’ role in promoting followers’ self-sanctions. This study shed light on the effect of leaders’ interactional justice on followers’ self-sanctions, embodied as mitigating employees’ moral disengagement. In addition, building on the underpinning framework of the interplay between personal and social sanctions (Bandura, 1999), we propose that two approaches complement each other to minimize employees’ misconduct—leaders’ interactional justice mitigates employees’ moral disengagement, and ethical leadership restrains employees’ moral disengagement from turning into deviance. In doing so, our study theoretically integrates the moral effects of interactional justice and ethical leadership and highlights how they can be studied together.

Second, our study provides a novel perspective that advances knowledge about how leaders can influence moral disengagement. For decades, despite the research of outcomes of moral disengagement has waxed, little is known regarding when moral disagreement induces undesirable behaviors (see Newman et al., 2020, as a review). Individuals who escape self-sanctions may not necessarily engage in misconducts, since the social sanctions are essential to be considered. As Bandura (1991, 1999) highlighted in his seminal work, moral action is regulated by the interplay of social and personal sanctions. Thus, this research begins to empirically explicate how pronounced social sanctions can suppress self-approval of deviance. By examining the effect of ethical leadership on the relationship between employees’ moral disengagement and deviance, we contribute a comprehensive understanding of when moral disengagement exerts influence.

Finally, we theorize the social sanction effect of ethical leadership and propose its moderating role, which provides a novel, distal mechanism beyond the social learning perspective that focuses on its main effects. It is well-documented that ethical leadership exerts a significant moral impact on employees’ normative behavior (Bedi et al., 2016; Peng and Kim, 2020). Nevertheless, the existing literature has predominately focused on observational learning, modeling explanations that the mechanisms are primarily captured by ethical leadership itself (Brown and Treviño, 2006). Drawing on social cognitive theory, this research proposes the social sanction effect of ethical leadership (Bandura, 1999). Ethical leadership increases employees’ sense of the “cost” of engaging in misconduct, which can prevent deviance even if employees have a deactivated moral self-regulation system via moral disengagement (Hirsh et al., 2018). Given this finding, we extend the understanding of how ethical leadership exerts moral influence and thus respond to the calls to investigate various pathways by which ethical leadership’s effects may emerge (Brown and Mitchell, 2010; Moore et al., 2019).

### Managerial implications

This research also has crucial practical implications. Although many organizations rely on supervisors to influence employees, regulating followers’ ethical behavior is resource-consuming for leaders and may cause resentment among employees (Owens et al., 2019). Against this background, this study suggests that interactional justice is essential for leaders to mitigate employees’ moral disengagement in routine work. Interactional justice behaviors are familiar to most people and straightforward to enact (Bies, 2015). Importantly, our research reveals that leaders’ interactional justice activates the employee’s moral self-regulation system to restrain misbehavior through self-sanctions. Accordingly, we suggest that organizations may reap benefits by reminding supervisors of the moral impact of interactional justice. Treating subordinates truthfully and respectfully is not only about a positive leader–member relationship but also mitigates subordinates’ dysfunctional behaviors that harm the interests of the whole organization.

Moreover, another critical strategy for leaders to curb followers’ deviance is promoting social sanctions. We suggest practitioners adopt the behavioral approaches of ethical leadership to prevent employees’ moral disengagement from turning into deviance that harms the organization and its members. Specifically, this research suggests that ethical leadership can activate social sanctions by specifying and promoting normatively appropriate behavior in the organization. Violating the rules that ethical leaders value runs the risk of losing various opportunities or being directly punished. Hence, when employees have found excuses to violate the rules, leaders should underscore the importance of moral codes, which increase the perceived “cost” of partaking in misbehaviors. As a result, severe social consequences highlighted by ethical leadership can constrain followers’ deviance even if they are morally disengaged.

### Limitation and future directions

Our research has several limitations. First, although our three-wave time-lagged research design offers a relatively robust argument for the causal ordering of the variables, we cannot unequivocally determine the direction of causality. Moreover, as mentioned in past research, the experience of interactional justice has daily variance (Matta et al., 2020). Future research could investigate the possibility of spiral relationships between interactional justice and deviance by using a longitudinal research design and further extend the understanding of the moral impact of leaders’ interactional justice at within-person level, such as how employees construe moral decisions when their leaders’ interactional justice has a high daily variance (Matta et al., 2020).

Accordingly, we believe that our finding provides an avenue for future theory-building and empirical research. First, might other forms of fairness have moral implications? Our research focuses on interactional justice because of its moral-laden principles (truth and human dignity, Bies, 2015). Nevertheless, research involving deontic models argues that each type of justice can produce deontic reactions (Folger and Glerum, 2015; O’Reilly et al., 2016) because fairness itself is grounded in the notion that “people should get the treatment that they deserve”. Thus, in addition to interactional justice, procedural justice and outcome justice may have an impact on individuals’ abstract moral reasoning as well, leading to a change in moral cognition and decision-making (Crawshaw et al., 2013).

Furthermore, in regard to the integration of fairness and ethics, might employees’ own moral characteristics influence their sensitivity toward leaders’ interactional justice? Past research indicates that individuals in different moral frameworks focus on different aspects of ethics, such as rules or human rights (Chiu et al., 1997). As a research future direction, we propose that such differences may influence the moral implication of interactional justice. Leaders’ interactional justice has a stronger impact on employees who have incremental moral beliefs because such employees are more confident about developing their moral virtues and being respectable (Owens et al., 2019). Existing research has revealed that employee entity moral beliefs are more easily influenced by ethical leadership because such employees inherently focus on the rules (Zhu et al., 2015). Moreover, future research could consider culture-based value as well, such as Zhongyong thinking (Liu et al., 2022).

## Conclusion

Drawing on social cognitive theory, this research integrates two behavioral approaches for regulating employees’ deviance. Leaders’ interactional justice restrains followers’ moral disengagement in a dyadic relationship whereas ethical leadership provides the collective rule of normative behavior, mitigating the effect of followers’ moral disengagement on deviance. Thus, our research contributes to the literature on fairness and behavioral ethics by identifying the similarities and distinctions of the moral impacts between interactional justice and ethical leadership and integrating these two approaches into one framework.

## Data availability statement

The raw data supporting the conclusions of this article will be made available by the authors, without undue reservation.

## Ethics statement

This study was reviewed and approved by the Internal Review Board of College of Business, Shanghai University of Finance and Economics. All patients/participants were informed about study procedures on the cover page of the questionnaire and provided their written informed consent to participate in this study.

## Author contributions

JL designed the study, collected the data, and revised the manuscript. HW wrote the draft of the manuscript. YC and ZC revised the manuscript. All authors contributed to the article and approved the submitted version.
